# Evolving evidence in the treatment of primary and recurrent posterior cruciate ligament injuries, part 1: anatomy, biomechanics and diagnostics

**DOI:** 10.1007/s00167-020-06357-y

**Published:** 2020-11-17

**Authors:** Philipp W. Winkler, Bálint Zsidai, Nyaluma N. Wagala, Jonathan D. Hughes, Alexandra Horvath, Eric Hamrin Senorski, Kristian Samuelsson, Volker Musahl

**Affiliations:** 1 Department for Orthopaedic Sports Medicine, Klinikum rechts der Isar, Technical University of Munich, Ismaninger Str. 22, 81675 Munich, Germany; 2 Department of Orthopaedic Surgery, UPMC Freddie Fu Sports Medicine Center, University of Pittsburgh, 3200 S. Water St, Pittsburgh, PA 15203 USA; 3 Department of Orthopaedics, Sahlgrenska University Hospital, Mölndal, Sweden; 4 Department of Internal Medicine and Clinical Nutrition, Institute of Medicine, Sahlgrenska Academy, University of Gothenburg, Gothenburg, Sweden; 5 Department of Health and Rehabilitation, Institute of Neuroscience and Physiology, Sahlgrenska Academy, University of Gothenburg, Gothenburg, Sweden; 6 Department of Orthopaedics, Institute of Clinical Sciences, Sahlgrenska Academy, University of Gothenburg, Gothenburg, Sweden

**Keywords:** Posterior cruciate ligament, PCL, Revision, Knee, Anatomy, Biomechanics, Posterior stress radiograph, Diagnostic workup

## Abstract

The posterior cruciate ligament (PCL) represents an intra-articular structure composed of two distinct bundles. Considering the anterior and posterior meniscofemoral ligaments, a total of four ligamentous fibre bundles of the posterior knee complex act synergistically to restrain posterior and rotatory tibial loads. Injury mechanisms associated with high-energy trauma and accompanying injury patterns may complicate the diagnostic evaluation and accuracy. Therefore, a thorough and systematic diagnostic workup is necessary to assess the severity of the PCL injury and to initiate an appropriate treatment approach. Since structural damage to the PCL occurs in more than one third of trauma patients experiencing acute knee injury with hemarthrosis, background knowledge for management of PCL injuries is important. In Part 1 of the evidence-based update on management of primary and recurrent PCL injuries, the anatomical, biomechanical, and diagnostic principles are presented. This paper aims to convey the anatomical and biomechanical knowledge needed for accurate diagnosis to facilitate subsequent decision-making in the treatment of PCL injuries.

**Level of evidence** V.

## Introduction

Posterior cruciate ligament (PCL) tears are severe injuries with devastating long-term effects for the knee joint. Despite a fairly rare estimated incidence of 1–6% for isolated PCL tears [7, 12, 36, 40, 66], one study showed that structural damage to the PCL occurs in up to 38% of trauma patients presenting with acute knee injuries with hemarthrosis [12]. Consequently, over 60% of PCL injuries are associated with additional capsuloligamentous lesions [12, 36, 40, 56, 67, 68]; combined PCL and posterolateral corner (PLC) injuries are predominant, with a prevalence of 15–42% among PCL injured patients [12, 36, 67]. Since males are more often involved in trauma, males are more commonly affected by PCL injuries than females, and the average age at the time of injury is 28–34 years [40, 56, 67, 68]. Given the larger cross-sectional area and the increased tensile strength of the PCL, compared to the anterior cruciate ligament (ACL) [23], PCL injuries are associated with high-energy trauma, as observed in motor vehicle and sport-related injuries [12, 40, 56, 67, 68]. However, PCL injuries are also associated with low-velocity and ultra-low-velocity knee dislocations occurring during daily activities and typically affect obese patients [5, 74].

Over the past decades, research has provided insights into the fundamentals of PCL anatomy and biomechanics [1, 3, 13, 23, 31, 55, 59]. Diagnostic tools have evolved, leading to improved accuracy in detecting isolated and combined PCL injuries. As a result, the spectrum of treatment approaches and the corresponding indications has expanded.

## Anatomy

**Fig. 1:**
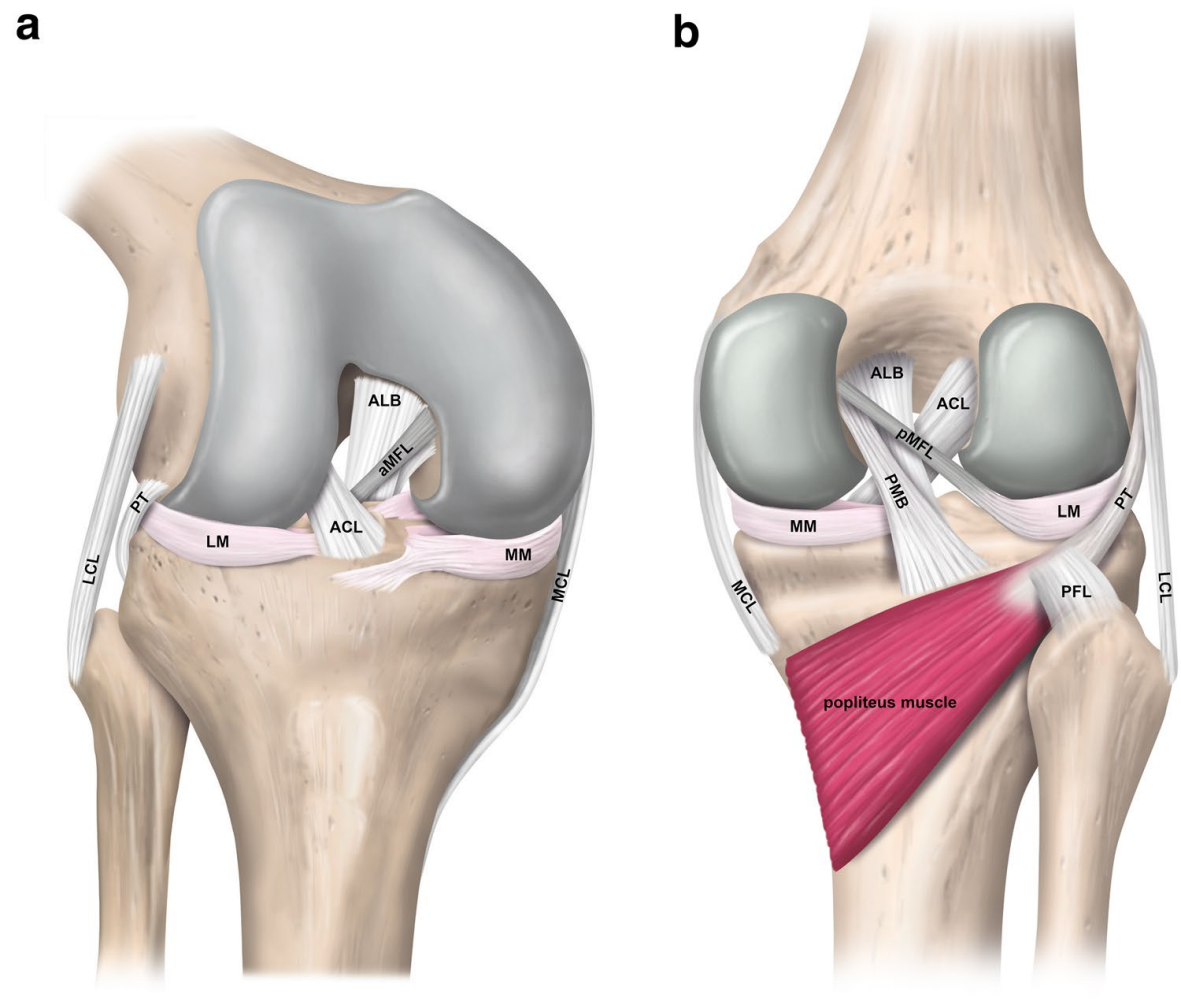
Schematic illustration of the anatomy of the posterior cruciate and the meniscofemoral ligaments. **a** Right knee from an antero-lateral view. **b** Right knee from a posterior view. *ACL* anterior cruciate ligament; *ALB* anterolateral bundle; *aMFL* anterior meniscofemoral ligament; *LCL* lateral collateral ligament; *LM* lateral meniscus; *MCL* medial collateral ligament; *MM* medial meniscus; *PFL* popliteofibular ligament; *PMB* posteromedial bundle; *pMFL* posterior meniscofemoral ligament; *PT* popliteus tendon

**Fig. 2:**
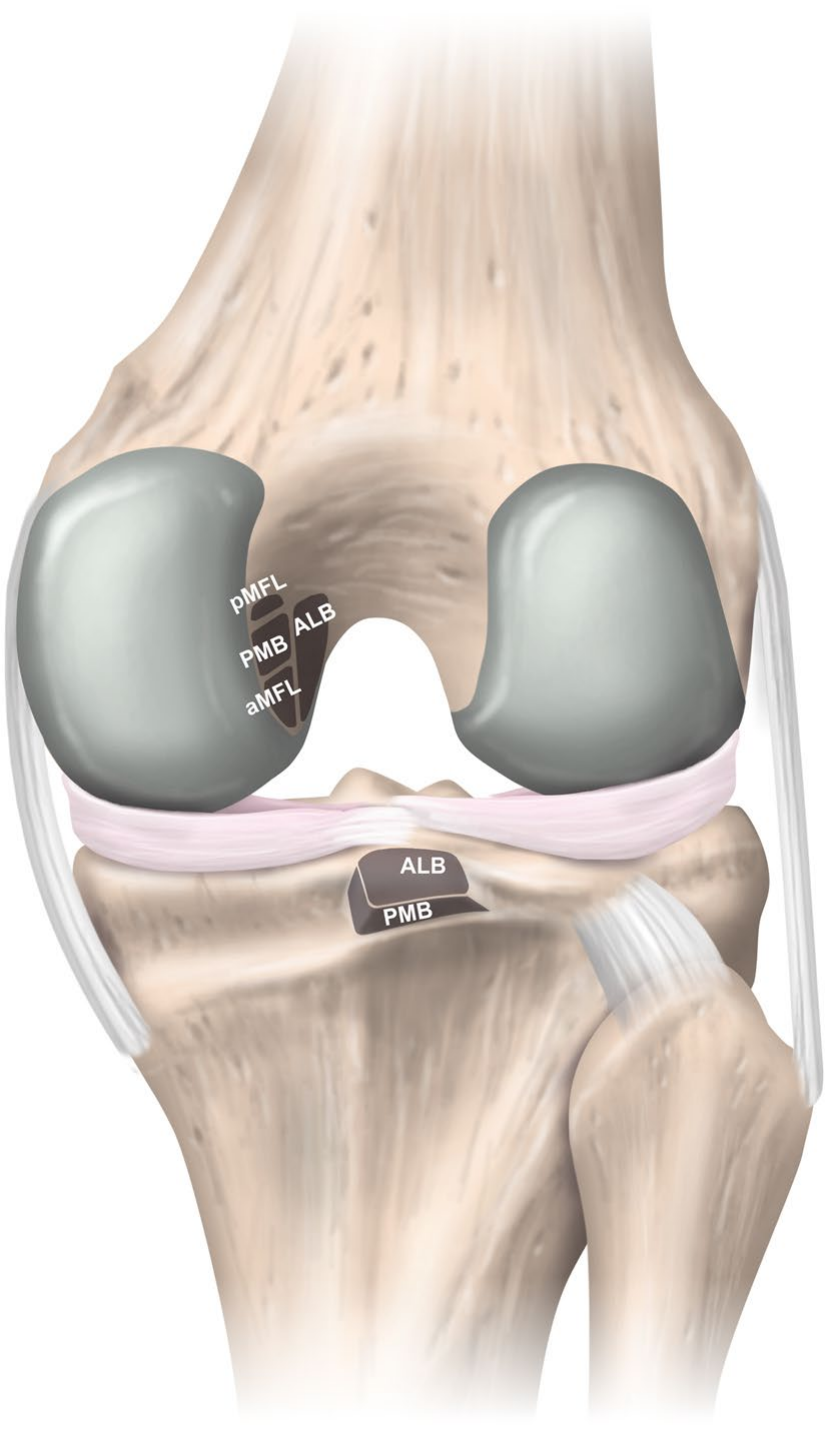
Schematic illustration of the insertional zones of the posterior cruciate ligament in the right knee (posterior view). *ALB* anterolateral bundle; *aMFL* anterior meniscofemoral ligament; *PMB* posteromedial bundle; *pMFL* posterior meniscofemoral ligament

With an average length of 36–38 mm and a mean cross-sectional area of 40–60 mm^2^ at the midsubstance level, the PCL is an intra-articular, extra-synovial ligamentous structure of the knee [23, 31, 55]. It is generally accepted and supported by numerous anatomical and biomechanical studies that the PCL consists of two distinct bundles (Fig. 1) [3, 71]. More prominent, larger in its cross-sectional area, and stronger against tensile stress is the anterolateral bundle (ALB). In contrast, the posteromedial bundle (PMB) represents a weaker and anatomically more diverse part of the PCL [3, 4, 23, 59]. The extensive half-moon shaped femoral attachment site is located on the lateral facet of the medial femoral condyle, involves anteriorly the roof of the intercondylar notch, is proximally bounded by the medial intercondylar ridge, reaches distally the margin of the articular cartilage of the medial femoral condyle, and thus covers an area of approximately 190–230 mm^2^ (Fig. 2) [3, 4, 17, 71]. Based on a modified quadrant method superimposed on the medial femoral condyle, on strict lateral radiographs, the centre of the femoral insertion site of the ALB is located at approximately 38–42% of the depth (measured from the anterior cartilage margin) and 13–16% of the height (measured from the roof of the intercondylar notch) of the medial femoral condyle along and perpendicular to the Blumensaat’s line, respectively [42, 52, 55]. The corresponding values for the PMB are 49–63% and 35–38%, respectively [42, 55]. According to a recent anatomic study, the midsubstance of the PCL appears to be flat with a mean width and thickness of approximately 13 mm and 5 mm, respectively [31]. The significantly smaller and trapezoidal-shaped tibial attachment site of the PCL covers an area of approximately 160–220 mm^2^ and is in close proximity to the posterior root of the medial and lateral menisci [3, 4, 17]. The centre of the tibial PCL attachment is located slightly distal to the articular surface in a sulcus termed the PCL facet, which is located between the medial and lateral tibial plateau condyle at approximately 50% (measured from the medial tibial border) of the tibial plateau’s total medial–lateral diameter [4, 17, 42, 50, 55, 71]. More specifically, the tibial footprint is bounded posteriorly by the champagne-glass drop-off, a bony landmark at the transitional zone to the popliteus muscle which can be consistently identified on lateral radiographs. Another pertinent bony landmark is called the bundle ridge, which separates the tibial attachment site of the ALB and PMB [4].

The meniscofemoral ligaments (MFLs) are an integral part of the posterior knee complex. Given the position of the two MFLs with respect to the PCL, a distinction is made between the anterior MFL (Ligament of Humphrey) and the posterior MFL (Ligament of Wrisberg). The anterior and posterior MFL can be observed in 20–75% and 70–100% of knees, respectively, and at least one MFL is present in more than 90% [4, 19, 51]. On the femoral side, the MFLs attach just proximal (posterior MFL) and distal (anterior MFL) to the femoral attachment of the PMB and exhibit a circular cross section (Fig. 2) [4, 23]. In spite of the detailed description of the femoral MFL insertion site, precise knowledge about the attachment on the posterior horn of the lateral meniscus is limited [3, 4, 23, 71]. In one study, a mean distance from the centre of the posterolateral meniscal root to the meniscal attachment of the anterior MFL and the posterior MFL of 6 mm and 12 mm, respectively, could be observed [2]. Some anatomical variations of the posterior MFL with menisco-tibial and tibio-femoral fibres have been demonstrated as well [31].

### Biomechanics

The ALB and PMB were thought to be reciprocally involved during knee flexion. However, recent investigations highlight the codominant characteristics of the two bundles throughout the knee’s range of motion (ROM) [1, 34, 57]. The resisting force against posterior tibial translation (PTT) provided by the ALB and PMB depends on the orientation and tension of the respective bundle. Since the length and orientation of the ALB and PMB behave reciprocally, neither bundle can be considered as the primary restraint against PTT at certain knee flexion angles, which is defined as codominance [1]. The magnitudes of force and strain exerted on the individual bundles of the PCL are largely influenced by knee flexion angle and type of activity [25]. Both bundles have been demonstrated to provide greater functional roles during flexion rather than extension, which is further supported by findings suggesting that the length of the ALB and PMB increases during knee flexion, both with and without application of a posteriorly directed force [57, 77].

Biomechanical investigations of the PCL have elucidated its primary role as providing restraint against PTT, with recent studies implicating its additional involvement as a secondary stabilizer against rotatory loads, particularly above 90° of knee flexion [32–34]. However, the extent of increased anterior–posterior (AP) and rotatory knee laxity depends on whether one or both PCL bundles are injured. One study comparing partially and completely transected PCL specimens with PCL intact specimens demonstrated that complete sectioning of the PCL results in higher magnitudes of PTT between flexion angles of 0° and 120°, as well as increased internal and external rotatory laxity between 90° and 120° [34]. Consistent findings have denoted that partial PCL injury may not have a clinically relevant effect with respect to PTT [21, 46]. However, an in vivo investigation of tibiofemoral laxity during functional activities in partially PCL injured patients demonstrated that the magnitude and timing of dynamic laxity differ among patients with a similar degree of static laxity. This finding further supports the notion of various patient-related factors contributing to functional instability following PCL injuries [16].

**Fig. 3:**
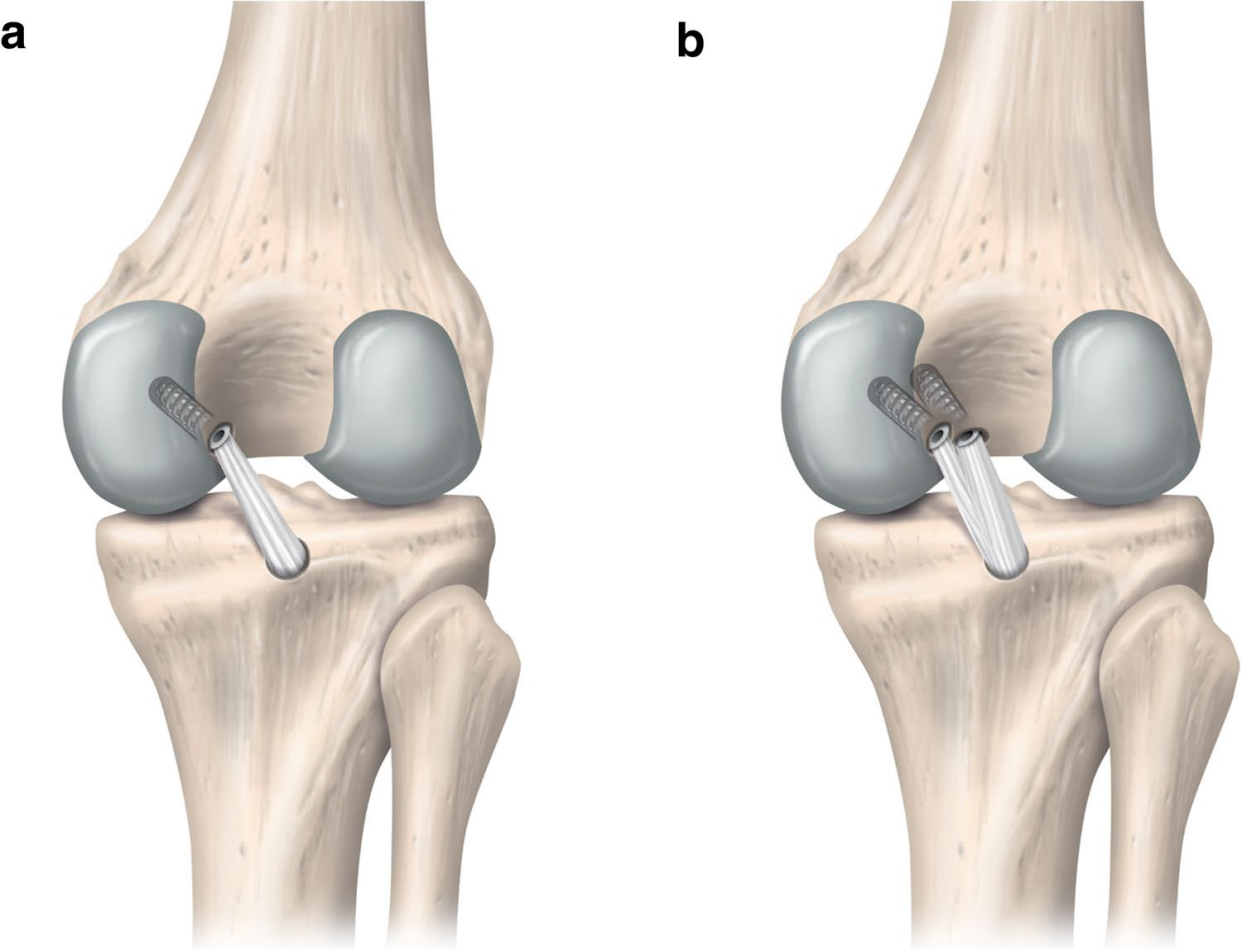
Right knee from a posterior view demonstrating single-bundle vs. double-bundle posterior cruciate ligament reconstruction (PCL-R). **a** Transtibial single-bundle PCL-R. **b** Transtibial double-bundle PCL-R

A considerable part of the current literature focuses on how to best restore native PCL biomechanics in a manner that translates to clinical success following operative treatment of PCL injured patients. Biomechanical studies have reported anatomic double-bundle (DB) PCL reconstruction (PCL-R) to provide superior restoration of knee laxity and kinematics compared to anatomic single-bundle (SB) reconstruction (Fig. 3) [21, 49, 78]. Anatomic DB PCL-R has been shown to be superior to anatomic SB PCL-R in providing restraint to PTT between 15° and 120°, and internal rotation above 90° of knee flexion [78]. Conversely, results published by another investigation have raised the concern that tensions exerted on the PMB graft fixed at 30° during DB PCL-R results in excessively restricted residual AP translation of the knee, while the SB technique provides a superior replication of the native knee laxity [47].

The structures of the PLC of the knee, including the lateral collateral ligament, popliteus tendon, and popliteofibular ligament (Fig. 1) have been identified as restraints against PTT, varus torque, and tibial external rotation and, therefore, act synergistically with the PCL [11, 15, 37, 72, 76]. Increased in situ forces of the intact PCL have been demonstrated in externally loaded, isolated PLC-deficient knees, indicating the importance of detecting and treating PLC injuries concurrently with PCL-R to prevent excessive graft stress [22, 48]. This is also supported by a recent meta-analysis reporting that treating combined PCL and PLC injured knees with isolated SB or DB PCL-R is not able to restore native knee kinematics [38]. It has been shown that the medial collateral ligament (MCL) complex and the posteromedial corner (PMC) contribute to translational and rotatory knee laxity in PCL deficiency [58, 62, 64]. Controversy exists whether the superficial MCL or the posterior oblique ligament acts primarily [58, 62]. Consequently, the PMC may play a major role in residual knee laxity and needs to be assessed during PCL-R.

### Diagnostic workup

A thorough and systematic diagnostic workup including patient history, standardized clinical knee examination, and imaging is essential for each patient to precisely identify PCL and concomitant ligamentous, meniscal, and cartilage injuries for appropriate and individualized treatment decision-making [8, 20, 79].

Obtaining detailed information about the patient's medical history, injury mechanism, chief complaint as well as social, professional, and athletic demands is crucial [20, 41, 45, 79]. Usually, patients presenting with PCL injuries are aged between 20 and 35 years and have a history of a high-energy trauma caused by a motor vehicle or sport-related injury [12, 40, 56, 68]. More precisely, a posterior directed force acting on the proximal tibia leads to posterior tibial translation, which ultimately causes a disruption of the PCL [45, 68]. Other injury mechanisms are hyperflexion and also hyperextension, which is associated with a proximal tear location and anterior tibial plateau compression fractures [13, 61, 77]. Excessive varus, valgus, internal, or external torque of the tibia are also injury mechanisms and may be related to concomitant injuries of the peripheral capsuloligamentous structures, menisci, and cartilage [45, 56, 68]. Clinically, patients primarily report pain or discomfort after an acute PCL injury. The pain commonly affects the patellofemoral, anteromedial, or posterior part of the knee and occurs during uphill or downhill walks. Unlike in ACL deficiency, patients with isolated PCL tears rarely report symptoms of instability. However, the perception of instability becomes more present in chronic and combined PCL injuries [41, 43, 45, 79]. Hemarthrosis in patients after acute knee injuries may be indicative of PCL tears [12, 36], which is why a standardized clinical examination based on the International Knee Documentation Committee Form (IKDC) is recommended in such cases. Clinical tests such as the posterior drawer test, dial test, reversed pivot-shift test, and the quadriceps active test should be conducted and assessed in conjunction with clinical signs such as the posterior sag sign, lower limb alignment, or a varus thrust to accurately identify all injured structures [9, 41, 45, 79]. Posterior and posterolateral laxity can be exaggerated by an underlying varus malalignment. Varus knees can be classified as primary-, double-, or triple-varus. While primary varus is caused by osseous malalignment and loss of the medial meniscus or articular cartilage, double- and triple varus additionally include lateral and posterolateral ligamentous deficiency, respectively [53]. As a result, a varus thrust may be observed during gait analysis, which is defined as a dynamically increasing varus alignment under weight-bearing conditions.

In the authors’ (PWW, VM) approach, the posterior drawer test is performed in internal, neutral, and external rotation of the tibia to assess additional injuries of the peripheral capsuloligamentous structures since biomechanical studies have shown that the structures of the PMC and PLC are secondary restraints against internal and external tibial torque, respectively [30, 35, 54, 58, 70, 75]. Furthermore, all PCL injuries are classified according to the classifications proposed by the American Medical Association [60], Hughston and colleagues [27, 28], and Harner et al. [20]. Grade I and II injuries are considered as partial tears involving ligament strain or individual fibre disruption, quantified by a PTT of < 5 mm and 5–10 mm, respectively. Grade III or complete PCL tears are defined by total ligament rupture, exhibiting gross instability with a PTT of > 10 mm [20]. All tests are performed bilaterally to evaluate side-to-side differences and are complemented by the assessment of generalized hyperlaxity based on the Beighton scoring system [6].

**Fig. 4:**
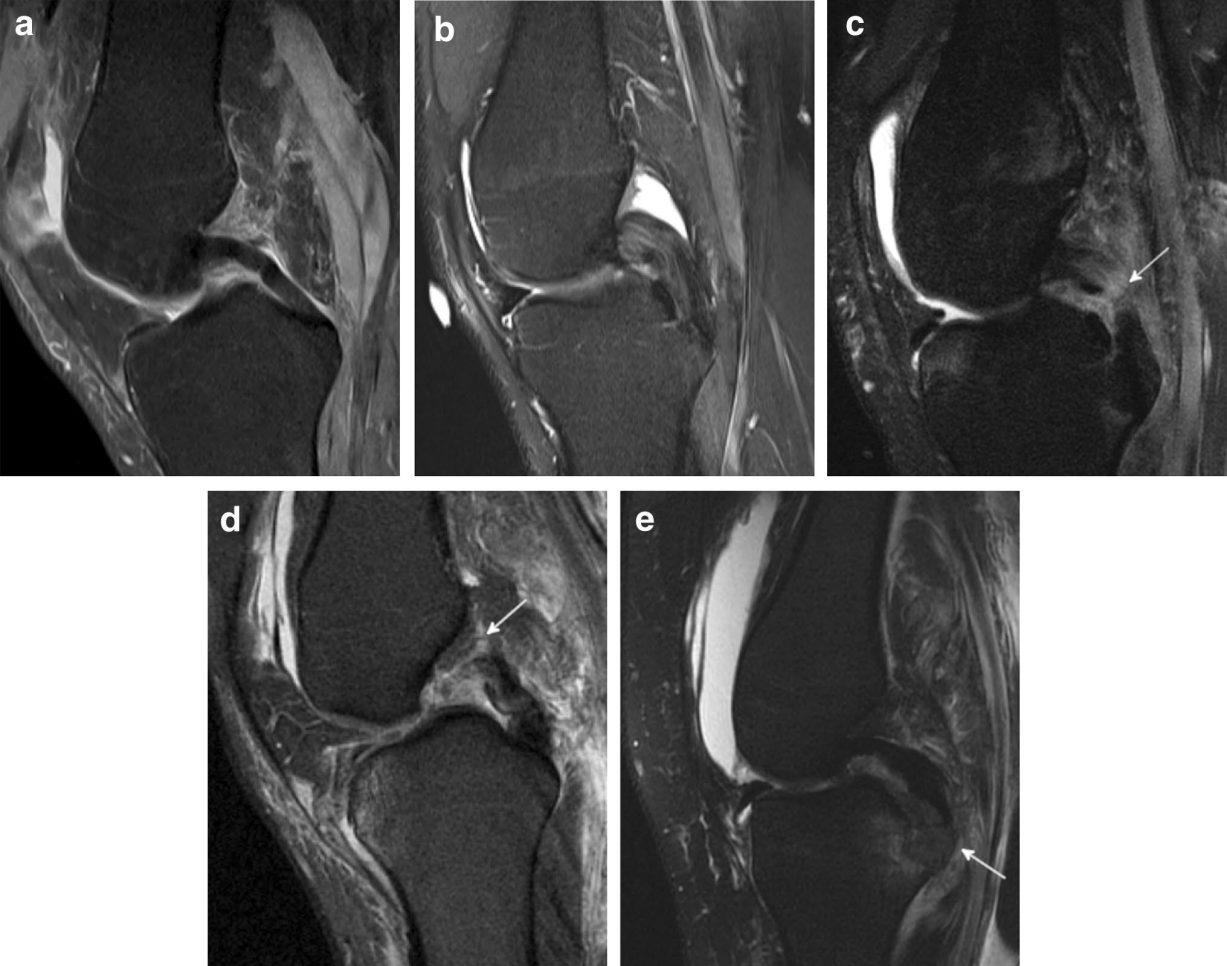
Sagittal T2-weighted magnetic resonance imaging scans of the posterior cruciate ligament (PCL). **a** Intact PCL. **b** Partial grade II PCL injury. Note the increased sagittal diameter of the PCL and the striated increased intraligamentary signal intensity. **c** Midsubstance grade III PCL injury. **d** Femoral grade III PCL injury. **e** Grade III tibial avulsion injury of the PCL. White arrow denotes injury site

Despite reliable clinical tests, magnetic resonance imaging (MRI) continues to be the most accurate diagnostic modality to identify isolated and combined PCL injuries (Fig. 4) [10, 18, 65]. Although the sensitivity and specificity of acute PCL injuries are reported to be up to 100%, this is not applicable for chronic and recurrent PCL injuries since scar formation or healing in a stretched, insufficient conformation may incorrectly portray an intact appearance of the PCL [10, 18, 69]. The AP diameter, as measured on sagittal T2-weighted MRI scans, of the native PCL has been shown to be ≤ 6 mm in 92% of patients without a history of knee trauma. In surgically confirmed PCL tears, the corresponding AP diameter was found to be ≥ 7 mm in 94% of patients. An increased AP diameter (≥ 7 mm) on sagittal T2-weighted images is, therefore, indicative for PCL injuries. Another sign of PCL tears is an increased intraligamentary signal intensity on fat-suppressed proton-density MR images, which may appear in a longitudinal striated configuration [65]. A buckled conformation of the PCL seen on sagittal MR images, mimics a slack appearance of the PCL and is caused by anterior displacement of the tibia. However, the buckled PCL sign is a secondary sign of ACL tears and should not be misinterpreted as a sign of PCL deficiency [63, 73, 80]. Specific bone bruise patterns are considered to be secondary signs of ligamentous knee injuries which additionally provide insights into the injury mechanism. Acute isolated PCL injuries and combined PCL and PLC injuries have been associated with a bone bruise in more than 80% of patients [14, 44]. More precisely, a bone bruise most frequently affects the anteromedial compartment, but depending on the concomitant injuries, it can also be observed in the lateral and patellofemoral compartment [14, 44]. Thus, bone bruise patterns in PCL injuries exhibit a certain diversity compared to ACL injuries.

**Fig. 5:**
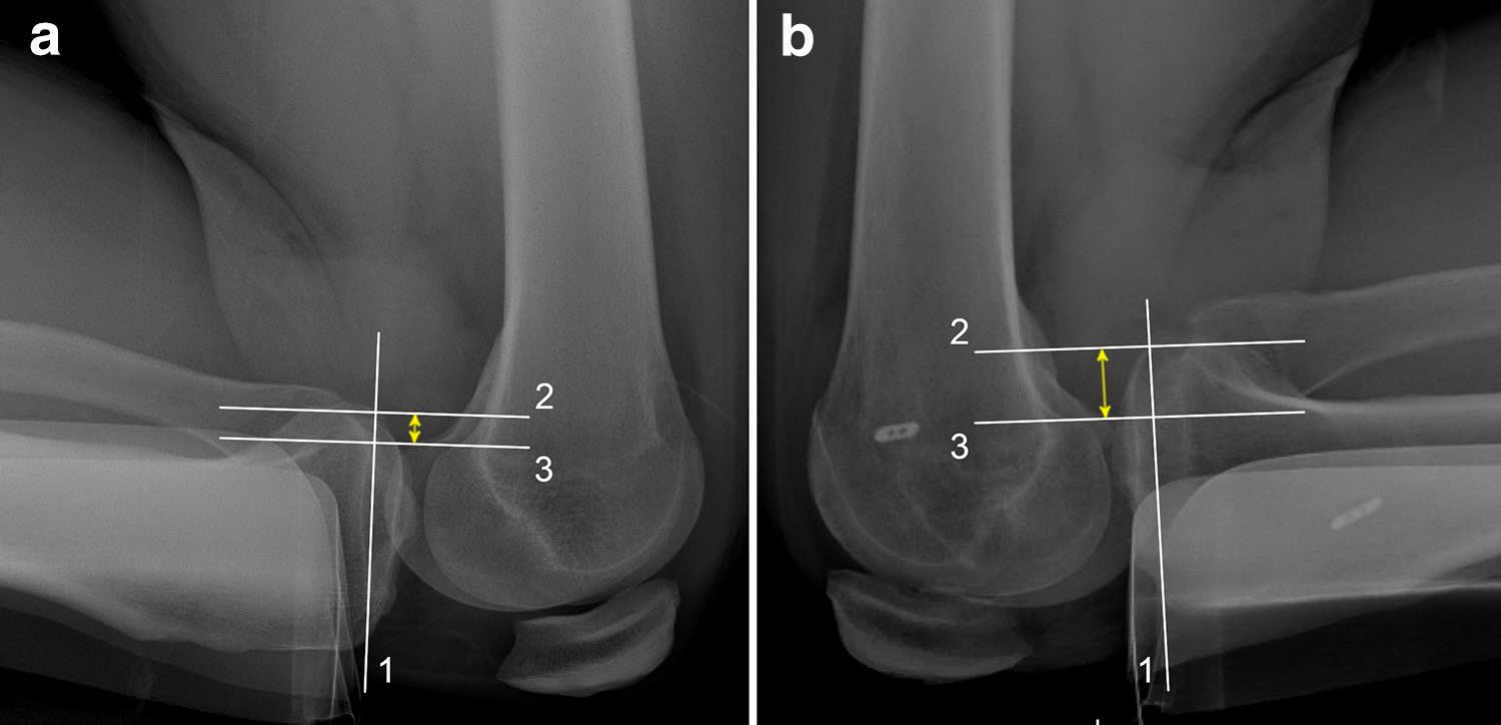
Posterior stress radiographs. Left, 8-mm posterior tibial translation (PTT) (**a**) and right, 18-mm PTT (**b**) quantified by lateral kneeling stress radiographs of a patient with failed posterior cruciate ligament reconstruction of the right knee. Posterior tibial translation is measured as the distance between line 2 and line 3 (yellow double-headed arrow). Line 1, tangential to the medial tibial plateau; Line 2, perpendicular to line 1 and crossing the midpoint between the posterior border of the medial and lateral tibial plateau; Line 3, parallel to line 2 crossing the midpoint between the medial and lateral femoral condyle

Quantification of PTT supports treatment decision-making and is essential for follow-up evaluation in operatively and non-operatively treated isolated and combined PCL injuries [41, 79]. The inter- and intrarater reliability, assessed by the intraclass correlation coefficients (ICC), for instrumented laxity measurement based on the KT-1000 arthrometer have been reported to be moderate (interrater ICC, 0.29–0.64; intrarater ICC, 0.59–0.79) for PCL-deficient and reconstructed patients [26]. Additionally, posterior sagittal stress radiographs have been found to be favourable in quantifying PTT compared to arthrometric measurements and manual testing [24]. Therefore, it is recommended to obtain stress radiographs for the quantification of PTT in addition to conventional AP and lateral radiographs. Different techniques for the acquisition of posterior sagittal stress radiographs and various methods for PTT measurement have been described [29, 39]. Given the differences in the time required for image acquisition, inconvenience to the patient (pain), reproducibility, and the measurement reliability, each acquisition and measurement technique has its advantages and disadvantages [29, 39]. The authors’ (PWW, VM) preferred technique is shown in Fig. 5. Additional acquisition of full leg or varus/valgus stress radiographs is recommended if lower limb malalignment or concomitant injuries of the PMC or PLC are clinically suspected [8].

## Conclusion

Anatomical studies have increased the awareness of the bundle configuration and insertion zones of the PCL and the close relationship to the MFLs. This is supplemented by biomechanical studies which have demonstrated the length and orientation change patterns of the individual PCL bundles and the resulting codominant and synergistic function. Specifically, the ALB is the larger and more important bundle and is most commonly reconstructed for single bundle PCL-R surgery. Double bundle reconstructions are biomechanically superior and may be used in cases with chronic high-grade posterior instability. Extended basic knowledge can be translated into improved diagnostic workup, thus facilitating an individual treatment approach.

## Abbreviations

ACL: Anterior cruciate ligament
ALB: Anterolateral bundle
AP: Anterior–posterior
DB: Double bundle
ICC: Intraclass correlation coefficients
IKDC: International Knee Documentation Committee
MCL: Medial collateral ligament
MFL: Meniscofemoral ligaments
MRI: Magnetic resonance imaging
PCL: Posterior cruciate ligament
PCL-R: Posterior cruciate ligament reconstruction
PLC: Posterolateral corner
PMB: Posteromedial bundle
PMC: Posteromedial corner
PTT: Posterior tibial translation
ROM: Range of motion
SB: Single bundle

## References

[1] Ahmad CS, Cohen ZA, Levine WN, Gardner TR, Ateshian GA, Mow VC (2003). Codominance of the individual posterior cruciate ligament bundles. An analysis of bundle lengths and orientation. Am J Sports Med.

[2] Aman ZS, DePhillipo NN, Storaci HW, Moatshe G, Chahla J, Engebretsen L (2019). Quantitative and qualitative assessment of posterolateral meniscal anatomy: defining the popliteal hiatus, popliteomeniscal fascicles, and the lateral meniscotibial ligament. Am J Sports Med.

[3] Amis AA, Gupte CM, Bull AM, Edwards A (2006). Anatomy of the posterior cruciate ligament and the meniscofemoral ligaments. Knee Surg Sports Traumatol Arthrosc.

[4] Anderson CJ, Ziegler CG, Wijdicks CA, Engebretsen L, LaPrade RF (2012). Arthroscopically pertinent anatomy of the anterolateral and posteromedial bundles of the posterior cruciate ligament. J Bone Joint Surg Am.

[5] Azar FM, Brandt JC, Miller RH, Phillips BB (2011). Ultra-low-velocity knee dislocations. Am J Sports Med.

[6] Beighton P, Solomon L, Soskolne CL (1973). Articular mobility in an African population. Ann Rheum Dis.

[7] Bollen S (2000). Epidemiology of knee injuries: diagnosis and triage. Br J Sports Med.

[8] Chahla J, Murray IR, Robinson J, Lagae K, Margheritini F, Fritsch B (2019). Posterolateral corner of the knee: an expert consensus statement on diagnosis, classification, treatment, and rehabilitation. Knee Surg Sports Traumatol Arthrosc.

[9] Daniel DM, Stone ML, Barnett P, Sachs R (1988). Use of the quadriceps active test to diagnose posterior cruciate-ligament disruption and measure posterior laxity of the knee. J Bone Joint Surg Am.

[10] DePhillipo NN, Cinque ME, Godin JA, Moatshe G, Chahla J, LaPrade RF (2018). Posterior tibial translation measurements on magnetic resonance imaging improve diagnostic sensitivity for chronic posterior cruciate ligament injuries and graft tears. Am J Sports Med.

[11] Domnick C, Frosch KH, Raschke MJ, Vogel N, Schulze M, von Glahn M (2017). Kinematics of different components of the posterolateral corner of the knee in the lateral collateral ligament-intact state: a human cadaveric study. Arthroscopy.

[12] Fanelli GC, Edson CJ (1995). Posterior cruciate ligament injuries in trauma patients: part II. Arthroscopy.

[13] Fox RJ, Harner CD, Sakane M, Carlin GJ, Woo SL (1998). Determination of the in situ forces in the human posterior cruciate ligament using robotic technology. A cadaveric study. Am J Sports Med.

[14] Geeslin AG, LaPrade RF (2010). Location of bone bruises and other osseous injuries associated with acute grade III isolated and combined posterolateral knee injuries. Am J Sports Med.

[15] Gollehon DL, Torzilli PA, Warren RF (1987). The role of the posterolateral and cruciate ligaments in the stability of the human knee. A biomechanical study. J Bone Joint Surg Am.

[16] Goyal K, Tashman S, Wang JH, Li K, Zhang X, Harner C (2012). In vivo analysis of the isolated posterior cruciate ligament-deficient knee during functional activities. Am J Sports Med.

[17] Greiner P, Magnussen RA, Lustig S, Demey G, Neyret P, Servien E (2011). Computed tomography evaluation of the femoral and tibial attachments of the posterior cruciate ligament in vitro. Knee Surg Sports Traumatol Arthrosc.

[18] Gross ML, Grover JS, Bassett LW, Seeger LL, Finerman GA (1992). Magnetic resonance imaging of the posterior cruciate ligament. Clinical use to improve diagnostic accuracy. Am J Sports Med.

[19] Gupte CM, Smith A, McDermott ID, Bull AM, Thomas RD, Amis AA (2002). Meniscofemoral ligaments revisited. Anatomical study, age correlation and clinical implications. J Bone Joint Surg Br.

[20] Harner CD, Höher J (1998). Evaluation and treatment of posterior cruciate ligament injuries. Am J Sports Med.

[21] Harner CD, Janaushek MA, Kanamori A, Yagi M, Vogrin TM, Woo SL (2000). Biomechanical analysis of a double-bundle posterior cruciate ligament reconstruction. Am J Sports Med.

[22] Harner CD, Vogrin TM, Höher J, Ma CB, Woo SL (2000). Biomechanical analysis of a posterior cruciate ligament reconstruction. Deficiency of the posterolateral structures as a cause of graft failure. Am J Sports Med.

[23] Harner CD, Xerogeanes JW, Livesay GA, Carlin GJ, Smith BA, Kusayama T (1995). The human posterior cruciate ligament complex: an interdisciplinary study. Ligament morphology and biomechanical evaluation. Am J Sports Med.

[24] Hewett TE, Noyes FR, Lee MD (1997). Diagnosis of complete and partial posterior cruciate ligament ruptures. Stress radiography compared with KT-1000 arthrometer and posterior drawer testing. Am J Sports Med.

[25] Hosseini Nasab SH, List R, Oberhofer K, Fucentese SF, Snedeker JG, Taylor WR (2016). Loading patterns of the posterior cruciate ligament in the healthy knee: a systematic review. PLoS ONE.

[26] Huber FE, Irrgang JJ, Harner C, Lephart S (1997). Intratester and intertester reliability of the KT-1000 arthrometer in the assessment of posterior laxity of the knee. Am J Sports Med.

[27] Hughston JC, Andrews JR, Cross MJ, Moschi A (1976). Classification of knee ligament instabilities. Part I. The medial compartment and cruciate ligaments. J Bone Joint Surg Am.

[28] Hughston JC, Andrews JR, Cross MJ, Moschi A (1976). Classification of knee ligament instabilities. Part II. The lateral compartment. J Bone Joint Surg Am.

[29] Jung TM, Reinhardt C, Scheffler SU, Weiler A (2006). Stress radiography to measure posterior cruciate ligament insufficiency: a comparison of five different techniques. Knee Surg Sports Traumatol Arthrosc.

[30] Kaneda Y, Moriya H, Takahashi K, Shimada Y, Tamaki T (1997). Experimental study on external tibial rotation of the knee. Am J Sports Med.

[31] Kato T, Śmigielski R, Ge Y, Zdanowicz U, Ciszek B, Ochi M (2018). Posterior cruciate ligament is twisted and flat structure: new prospective on anatomical morphology. Knee Surg Sports Traumatol Arthrosc.

[32] Kennedy NI, LaPrade RF, Goldsmith MT, Faucett SC, Rasmussen MT, Coatney GA (2014). Posterior cruciate ligament graft fixation angles, part 1: biomechanical evaluation for anatomic single-bundle reconstruction. Am J Sports Med.

[33] Kennedy NI, LaPrade RF, Goldsmith MT, Faucett SC, Rasmussen MT, Coatney GA (2014). Posterior cruciate ligament graft fixation angles, part 2: biomechanical evaluation for anatomic double-bundle reconstruction. Am J Sports Med.

[34] Kennedy NI, Wijdicks CA, Goldsmith MT, Michalski MP, Devitt BM, Årøen A (2013). Kinematic analysis of the posterior cruciate ligament, part 1: the individual and collective function of the anterolateral and posteromedial bundles. Am J Sports Med.

[35] Kittl C, Becker DK, Raschke MJ, Müller M, Wierer G, Domnick C (2019). Dynamic restraints of the medial side of the knee: the semimembranosus corner revisited. Am J Sports Med.

[36] LaPrade RF, Wentorf FA, Fritts H, Gundry C, Hightower CD (2007). A prospective magnetic resonance imaging study of the incidence of posterolateral and multiple ligament injuries in acute knee injuries presenting with a hemarthrosis. Arthroscopy.

[37] LaPrade RF, Wozniczka JK, Stellmaker MP, Wijdicks CA (2010). Analysis of the static function of the popliteus tendon and evaluation of an anatomic reconstruction: the “fifth ligament” of the knee. Am J Sports Med.

[38] Lee DY, Park YJ, Kim DH, Kim HJ, Nam DC, Park JS (2018). The role of isolated posterior cruciate ligament reconstruction in knees with combined posterior cruciate ligament and posterolateral complex injury. Knee Surg Sports Traumatol Arthrosc.

[39] Lee YS, Han SH, Jo J, Kwak KS, Nha KW, Kim JH (2011). Comparison of 5 different methods for measuring stress radiographs to improve reproducibility during the evaluation of knee instability. Am J Sports Med.

[40] Lind M, Nielsen TG, Behrndtz K (2018). Both isolated and multi-ligament posterior cruciate ligament reconstruction results in improved subjective outcome: results from the danish knee ligament reconstruction registry. Knee Surg Sports Traumatol Arthrosc.

[41] Lopez-Vidriero E, Simon DA, Johnson DH (2010). Initial evaluation of posterior cruciate ligament injuries: history, physical examination, imaging studies, surgical and nonsurgical indications. Sports Med Arthrosc Rev.

[42] Lorenz S, Elser F, Brucker PU, Obst T, Imhoff AB (2009). Radiological evaluation of the anterolateral and posteromedial bundle insertion sites of the posterior cruciate ligament. Knee Surg Sports Traumatol Arthrosc.

[43] MacGillivray JD, Stein BE, Park M, Allen AA, Wickiewicz TL, Warren RF (2006). Comparison of tibial inlay versus transtibial techniques for isolated posterior cruciate ligament reconstruction: minimum 2-year follow-up. Arthroscopy.

[44] Mair SD, Schlegel TF, Gill TJ, Hawkins RJ, Steadman JR (2004). Incidence and location of bone bruises after acute posterior cruciate ligament injury. Am J Sports Med.

[45] Margheritini F, Mariani PP (2003). Diagnostic evaluation of posterior cruciate ligament injuries. Knee Surg Sports Traumatol Arthrosc.

[46] Markolf KL, Feeley BT, Tejwani SG, Martin DE, McAllister DR (2006). Changes in knee laxity and ligament force after sectioning the posteromedial bundle of the posterior cruciate ligament. Arthroscopy.

[47] Markolf KL, Jackson SR, McAllister DR (2010). Single- versus double-bundle posterior cruciate ligament reconstruction: effects of femoral tunnel separation. Am J Sports Med.

[48] Markolf KL, Wascher DC, Finerman GA (1993). Direct in vitro measurement of forces in the cruciate ligaments. Part II: the effect of section of the posterolateral structures. J Bone Joint Surg Am.

[49] Milles JL, Nuelle CW, Pfeiffer F, Stannard JP, Smith P, Kfuri M (2017). Biomechanical comparison: single-bundle versus double-bundle posterior cruciate ligament reconstruction techniques. J Knee Surg.

[50] Moorman CT, Murphy Zane MS, Bansai S, Cina SJ, Wickiewicz TL, Warren RF (2008). Tibial insertion of the posterior cruciate ligament: a sagittal plane analysis using gross, histologic, and radiographic methods. Arthroscopy.

[51] Nagasaki S, Ohkoshi Y, Yamamoto K, Ebata W, Imabuchi R, Nishiike J (2006). The incidence and cross-sectional area of the meniscofemoral ligament. Am J Sports Med.

[52] Narvy SJ, Pearl M, Vrla M, Yi A, Hatch GF (2015). Anatomy of the femoral footprint of the posterior cruciate ligament: a systematic review. Arthroscopy.

[53] Noyes FR, Barber-Westin SD, Hewett TE (2000). High tibial osteotomy and ligament reconstruction for varus angulated anterior cruciate ligament-deficient knees. Am J Sports Med.

[54] Noyes FR, Stowers SF, Grood ES, Cummings J, VanGinkel LA (1993). Posterior subluxations of the medial and lateral tibiofemoral compartments. An in vitro ligament sectioning study in cadaveric knees. Am J Sports Med.

[55] Osti M, Tschann P, Künzel KH, Benedetto KP (2012). Anatomic characteristics and radiographic references of the anterolateral and posteromedial bundles of the posterior cruciate ligament. Am J Sports Med.

[56] Owesen C, Sandven-Thrane S, Lind M, Forssblad M, Granan LP, Årøen A (2017). Epidemiology of surgically treated posterior cruciate ligament injuries in Scandinavia. Knee Surg Sports Traumatol Arthrosc.

[57] Papannagari R, DeFrate LE, Nha KW, Moses JM, Moussa M, Gill TJ (2007). Function of posterior cruciate ligament bundles during in vivo knee flexion. Am J Sports Med.

[58] Petersen W, Loerch S, Schanz S, Raschke M, Zantop T (2008). The role of the posterior oblique ligament in controlling posterior tibial translation in the posterior cruciate ligament-deficient knee. Am J Sports Med.

[59] Race A, Amis AA (1994). The mechanical properties of the two bundles of the human posterior cruciate ligament. J Biomech.

[60] Rachun A (1968). Standard nomenclature of athletic injuries.

[61] Ringler MD, Shotts EE, Collins MS, Howe BM (2016). Intra-articular pathology associated with isolated posterior cruciate ligament injury on MRI. Skeletal Radiol.

[62] Ritchie JR, Bergfeld JA, Kambic H, Manning T (1998). Isolated sectioning of the medial and posteromedial capsular ligaments in the posterior cruciate ligament-deficient knee. Influence on posterior tibial translation. Am J Sports Med.

[63] Robertson PL, Schweitzer ME, Bartolozzi AR, Ugoni A (1994). Anterior cruciate ligament tears: evaluation of multiple signs with MR imaging. Radiology.

[64] Robinson JR, Bull AM, Thomas RR, Amis AA (2006). The role of the medial collateral ligament and posteromedial capsule in controlling knee laxity. Am J Sports Med.

[65] Rodriguez W, Vinson EN, Helms CA, Toth AP (2008). MRI appearance of posterior cruciate ligament tears. AJR Am J Roentgenol.

[66] Sanders TL, Pareek A, Barrett IJ, Kremers HM, Bryan AJ, Stuart MJ (2017). Incidence and long-term follow-up of isolated posterior cruciate ligament tears. Knee Surg Sports Traumatol Arthrosc.

[67] Schlumberger M, Schuster P, Eichinger M, Mayer P, Mayr R, Immendörfer M (2020). Posterior cruciate ligament lesions are mainly present as combined lesions even in sports injuries. Knee Surg Sports Traumatol Arthrosc.

[68] Schulz MS, Russe K, Weiler A, Eichhorn HJ, Strobel MJ (2003). Epidemiology of posterior cruciate ligament injuries. Arch Orthop Trauma Surg.

[69] Servant CT, Ramos JP, Thomas NP (2004). The accuracy of magnetic resonance imaging in diagnosing chronic posterior cruciate ligament injury. Knee.

[70] Strauss EJ, Ishak C, Inzerillo C, Walsh M, Yildirim G, Walker P (2007). Effect of tibial positioning on the diagnosis of posterolateral rotatory instability in the posterior cruciate ligament-deficient knee. Br J Sports Med.

[71] Takahashi M, Matsubara T, Di M, Suzuki D, Nagano A (2006). Anatomical study of the femoral and tibial insertions of the anterolateral and posteromedial bundles of human posterior cruciate ligament. Knee Surg Sports Traumatol Arthrosc.

[72] Thaunat M, Pioger C, Chatellard R, Conteduca J, Khaleel A, Sonnery-Cottet B (2014). The arcuate ligament revisited: role of the posterolateral structures in providing static stability in the knee joint. Knee Surg Sports Traumatol Arthrosc.

[73] Vahey TN, Hunt JE, Shelbourne KD (1993). Anterior translocation of the tibia at MR imaging: a secondary sign of anterior cruciate ligament tear. Radiology.

[74] Vaidya R, Roth M, Nanavati D, Prince M, Sethi A (2015). Low-velocity knee dislocations in obese and morbidly obese patients. Orthop J Sports Med.

[75] Vap AR, Schon JM, Moatshe G, Cruz RS, Brady AW, Dornan GJ (2017). The role of the peripheral passive rotation stabilizers of the knee with intact collateral and cruciate ligaments: a biomechanical study. Orthop J Sports Med.

[76] Veltri DM, Deng XH, Torzilli PA, Warren RF, Maynard MJ (1995). The role of the cruciate and posterolateral ligaments in stability of the knee. A biomechanical study. Am J Sports Med.

[77] Wang JH, Kato Y, Ingham SJ, Maeyama A, Linde-Rosen M, Smolinski P (2014). Effects of knee flexion angle and loading conditions on the end-to-end distance of the posterior cruciate ligament: a comparison of the roles of the anterolateral and posteromedial bundles. Am J Sports Med.

[78] Wijdicks CA, Kennedy NI, Goldsmith MT, Devitt BM, Michalski MP, Årøen A (2013). Kinematic analysis of the posterior cruciate ligament, part 2: a comparison of anatomic single- versus double-bundle reconstruction. Am J Sports Med.

[79] Wind WM, Bergfeld JA, Parker RD (2004). Evaluation and treatment of posterior cruciate ligament injuries: revisited. Am J Sports Med.

[80] Yoo JD, Lim HM (2012). Morphologic changes of the posterior cruciate ligament on magnetic resonance imaging before and after reconstruction of chronic anterior cruciate ligament ruptures. Knee Surg Relat Res.

